# 血液病患者造血干细胞移植后血流感染及耐碳青霉烯类革兰阴性杆菌定植的重点菌群监测和临床分析

**DOI:** 10.3760/cma.j.cn121090-20230731-00040

**Published:** 2024-02

**Authors:** 奥 张, 晨静 千, 若文 韦, 姗 姜, 峻 方, 威 石, 凌辉 夏

**Affiliations:** 华中科技大学同济医学院附属协和医院血液科，武汉 430022 Department of Hematology, Union Hospital, Tongji Medical College, Huazhong University of Science and Technology, Wuhan 430022, China

**Keywords:** 造血干细胞移植, 血流感染, 肛周定植, 耐碳青霉烯类革兰阴性杆菌, 耐碳青霉烯肠杆菌, Hematopoietic stem cell transplantation, Bloodstream infection, Perianal colonization, Carbapenem-resistant organism, Carbapenem-resistant Enterobacteriaceae

## Abstract

**目的:**

探讨造血干细胞移植（HSCT）后感染和定植的病原菌分布及临床特征，以期为血液病患者移植后并发感染的重点菌群监测与临床诊疗提供初步研究依据。

**方法:**

回顾性分析2018年1月至2022年12月华中科技大学同济医学院附属协和医院血液科190例微生物检测［革兰阴性菌（G^−^菌）血培养和/或肛拭子耐碳青霉烯类革兰阴性菌（CRO）筛查］阳性患者的临床资料，根据患者检测结果分为血培阳组、肛拭阳组、双阳组。统计分析比较三组患者情况。

**结果:**

63例发生G^−^菌血流感染（BSI）的患者分离病原菌排名前四位的为大肠埃希菌（28株，43.75％）、肺炎克雷伯菌（26株，40.63％）、铜绿假单胞菌（3株，4.69％）和阴沟肠杆菌（3株，4.69％）。147例发生CRO肛周定植的患者分离病原菌排名前三位的为耐碳青霉烯肺炎克雷伯菌（58株，32.58％）、耐碳青霉烯大肠埃希菌（49株，27.53％）、耐碳青霉烯阴沟肠杆菌（20株，11.24％）。双阳组患者的3年无病生存（DFS）率和总生存（OS）率与血培阳组和肛拭阳组相比显著降低（DFS：35.6％对53.7％对68.6％，*P*＝0.001；OS：44.4％对62.4％对76.9％，*P*<0.001），非复发死亡率（NRM）显著增高（50.0％对34.9％对10.6％，*P*<0.001）。血小板未植入和发生BSI为NRM的独立危险因素（*P*<0.001）。加用多黏菌素和（或）头孢他啶阿维巴坦后使用天数>7 d为NRM的独立保护因素（*P*＝0.035）。

**结论:**

发生BSI会显著增加血液病患者HSCT后的NRM；CRO定植加入血会严重影响其DFS和OS。

## Abstract (en)

**Objective:**

To investigate the distribution and clinical characteristics of pathogenic bacteria following hematopoietic stem cell transplantation （HSCT）, as well as to provide a preliminary research foundation for key microbial monitoring, and clinical diagnosis and treatment of infections after HSCT in hematological patients.

**Methods:**

We retrospectively analyzed the clinical data of 190 patients who tested positive for microbial testing ［G-bacteria blood culture and/or carbapenem-resistant organism（CRO） screening of perianal swabs］ at our center from January 2018 to December 2022. Patients were divided into blood culture positive, perianal swab positive, and double positive groups based on the testing results. The three patient groups underwent statistical analysis and comparison.

**Results:**

The top four pathogenic bacteria isolated from sixty-three patients with G-bacteria bloodstream infection（BSI） were Escherichia coli（28 strains, 43.75％）, Klebsiella pneumonia（26 strains, 40.63％）, Pseudomonas aeruginosa （3 strains, 4.69％）, and Enterobacter cloacae （3 strains, 4.69％）. The top three pathogenic bacteria isolated from 147 patients with CRO perianal colonization were carbapenem-resistant Klebsiella pneumoniae （58 strains, 32.58％）, carbapenem-resistant Escherichia coli （49 strains, 27.53％）, and carbapenem-resistant Enterobacter cloacae （20 strains, 11.24％）. The 3-year disease-free survival（DFS ）and overall survival（OS） of double positive group patients were significantly lower compared to those in the blood culture and perianal swab positive groups （DFS: 35.6％ *vs* 53.7％ *vs* 68.6％, *P*＝0.001; OS: 44.4％ *vs* 62.4％ *vs* 76.9％, *P*<0.001）, while non-relapse mortality（NRM） was significantly higher （50.0％ *vs* 34.9％ *vs* 10.6％, *P*<0.001）. Failed engraftment of platelets and BSI are independent risk factors for NRM（*P*<0.001）. Using polymyxin and/or ceftazidime-avibactam for more than 7 days is an independent protective factor for NRM （*P*＝0.035）.

**Conclusion:**

This study suggests that the occurrence of BSI significantly increases the NRM after HSCT in patients with hematological diseases; CRO colonization into the bloodstream has a significant impact on the DFS and OS of HSCT patients.

造血干细胞移植（HSCT）目前仍是多种血液系统疾病的首选治疗方法[1]。由于血液疾病本身特点以及在进行移植治疗时，放/化疗预处理及预防移植物抗宿主病（GVHD）所用的免疫抑制剂，患者免疫功能严重受损[2]，移植后发生感染的风险增高[3]。在我国，革兰阴性菌（G^−^菌）感染的发生率占65.7％～73.0％，远超革兰阳性菌感染[4]。耐碳青霉烯类革兰阴性菌（CRO）被WHO和美国疾病控制与预防中心定为首要危险等级[5]。耐碳青霉烯肠杆菌（CRE）是临床常见的CRO，随着耐碳青霉烯类药物的广泛使用，CRE感染发生率逐年升高[6]。多项研究表明，血液病患者CRE入血引起血流感染（bloodstream infection, BSI）导致的死亡率高达51％～65％[7]。因此，提早筛查出CRO定植并预防BSI，对于改善血液病患者的预后具有重要意义。本研究我们回顾性分析了190例在我院行HSCT且微生物检测（G^−^菌血培养、肛拭子CRO筛查）阳性的病例资料，旨在为血液病患者移植后并发感染的重点菌群监测与临床诊疗提供初步的研究参考。

## 病例与方法

一、病例选择

本研究为回顾性观察性研究，2018年1月至2022年12月我中心共有1 187例患者进行微生物检测（G^−^菌血培养、肛拭子CRO筛查），其中190例（16.01％）患者检测结果阳性，纳入本研究。190例阳性患者中，43例仅G^−^菌血培养阳性（血培阳组），127例仅肛拭子CRO筛查阳性（肛拭阳组），20例两项检测结果均阳性（双阳组）。

二、预处理方案

168例行异基因造血干细胞移植（allo-HSCT）的患者中，采用基于白消安（Bu）的预处理145例（86.31％），基于全身照射（TBI）或全骨髓照射（TMI）的预处理13例（7.74％），氟达拉滨（Flu）+环磷酰胺（Cy）+抗胸腺/淋巴细胞球蛋白（ATG/ALG）的预处理10例（5.95％）。21例行自体移植（auto-HSCT）的患者中，均采用以马法兰（Mel）为主的预处理方案。

三、GVHD预防

168例行allo-HSCT的患者中，66例（39.29％）以环孢素A（CsA）为主，102例（60.71％）以他克莫司（FK506）为主，联合短程甲氨蝶呤（MTX）±霉酚酸酯（MMF）±巴利昔单抗（CD25）±ATG/ALG进行预防。

四、病原菌分离和鉴定

所有病原菌均分离自患者血液或肛周皮肤。在院期间，患者体温≥38 °C或发生低热伴畏寒、寒战，在使用/调整抗菌药前均用标准无菌法从外周静脉取血，连续抽取2次，进行需氧/厌氧菌培养[8]。我中心移植患者每7 d均会进行一次肛拭子CRO筛查。送检的标本由我院检验医师在临床微生物实验室中采用phoenix-100 全自动细菌鉴定系统（BD，美国）进行分离及鉴定。同一患者28 d内通过血培养或肛拭子检测出的同一病原菌仅纳入首次分离菌株时间进行分析[9]。

五、研究终点及定义

非复发死亡（NRM）定义为首次移植回输至因血液病复发以外原因引起死亡。急性移植物抗宿主病（aGVHD）参照Glucksberg标准[10]–[11]，慢性移植物抗宿主病（cGVHD）参照2014年美国国立卫生院标准[12]。造血重建、无病生存（DFS）及总体生存（OS）定义参照文献[13]标准。本研究主要终点为NRM。随访截至2023年6月15日。

六、统计学处理

采用SPSS 26.0及R 4.6.2进行数据分析。计量资料比较采用非参数检验（不符合正态分布），计数资料比较采用卡方检验或Fisher精确概率法。采用Bonferroni法进行多重检验校正。DFS、OS使用Kaplan-Meier法描述其分布。造血重建、GVHD、累积复发率（CIR）、NRM行竞争风险分析。采用Cox回归行单因素及多因素分析，单因素分析*P*<0.05的变量纳入多因素分析。双侧*P*<0.05为差异有统计学意义。

## 结果

一、一般资料

190例患者中，男102例（53.68％），女88例（46.32％），中位年龄为33（14～72）岁。血培阳组、肛拭阳组、双阳组患者的基线特征差异均无统计学意义（均*P*>0.05）（表1）。

**表1:** 190例造血干细胞移植后微生物检测阳性患者临床特征比较

临床特征	血培阳组（43例）	肛拭阳组（127例）	双阳组（20例）	*P*值
性别[例（%）]				0.725
男	28（65.12）	75（59.06）	13（65.00）	
女	15（34.88）	52（40.94）	7（35.00）	
年龄[岁，*M*（范围）]	41（14~62）	33（14~72）	32（15~55）	0.306
疾病类型[例（%）]				0.903
恶性血液病	37（86.05）	110（86.61）	18（90.00）	
急性白血病	20（46.51）	83（65.35）	14（70.00）	
慢性白血病	0（0）	1（0.79）	0（0）	
骨髓增生异常综合征	6（13.95）	10（7.87）	1（5.0）	
非霍奇金淋巴瘤	8（18.60）	12（9.45）	2（10.00）	
多发性骨髓瘤	3（6.98）	4（3.15）	1（5.0）	
非恶性血液病	6（13.95）	17（13.39）	2（10.00）	
再生障碍性贫血	5（11.63）	17（13.39）	2（10.00）	
阵发性睡眠性血红蛋白尿症	1（2.33）	0（0）	0（0）	
移植类型[例（%）]				0.069
异基因	34（79.97）	117（92.13）	17（85.00）	
自体	9（20.93）	9（7.08）	3（15.00）	
脐带血	0（0）	1（0.79）	0（0）	
供者类型[例（%）]				0.067
亲缘全相合	4（11.77）	35（29.91）	2（11.76）	
单倍体	28（82.35）	80（68.38）	15（88.24）	
无关供者	2（5.88）	2（1.71）	0（0）	
有核细胞输注量[×10^8^/kg，*M*（范围）]	12.80（3.31~24.70）	12.50（5.00~37.93）	11.67（2.88~20.60）	0.927
CD34^+^细胞输注量[×10^6^/kg，*M*（范围）]	6.16（2.26~15.50）	6.11（2.05~23.21）	6.89（2.35~12.10）	0.920

注 血培阳组：革兰阴性菌血培养阳性；肛拭阳组：肛拭子耐碳青霉烯类革兰阴性菌阳性；双阳组：两项检测结果均阳性

二、病原菌分布

63例（33.16％）发生G^−^菌BSI的患者共分离出64株病原菌，排名前4位的为大肠埃希菌（28株，43.75％）、肺炎克雷伯菌（26株，40.63％）、铜绿假单胞菌（3株，4.69％）和阴沟肠杆菌（3株，4.69％）。

147例（77.37％）发生CRO肛周定植的患者分离出178株病原菌，排名前3位的为耐碳青霉烯肺炎克雷伯菌（58株，32.58％）、耐碳青霉烯大肠埃希菌（49株，27.53％）、耐碳青霉烯阴沟肠杆菌（20株，11.24％）。

三、造血重建与GVHD发生情况

三组患者中性粒细胞中位植入时间差异无统计学意义（*P*＝0.175），肛拭阳组血小板中位植入时间短于血培阳组和双阳组（*P*<0.001）。双阳组与血培阳组和肛拭阳组相比+28 d粒系植入率和血小板植入率最低（*P*值分别为0.003、<0.001）。三组患者+100 dⅠ～Ⅱ度、Ⅲ～Ⅳ度aGVHD发生率和3年总cGVHD发生率差异均无统计学意义（均*P*>0.05），肛拭阳组3年中重度cGVHD发生率最高（*P*＝0.024）（表2）。

**表2:** 造血干细胞移植后微生物检测阳性患者造血重建与GVHD累积发生率比较

临床特征	血培阳组（43例）	肛拭阳组（127例）	双阳组（20例）	*P*值
中性粒细胞植入				
植入例数[例（%）]	39（90.70）	123（96.85）	11（55.00）	<0.001
植入时间[d，*M*（范围）]	13（9~24）	12（8~40）	12（10~23）	0.175
+28 d植入率[%（95% *CI*）]	90.70（90.25~91.15）	93.70（93.60~93.80）	55.00（52.12~57.88）	0.003
血小板植入				
植入例数[例（%）]	36（83.72）	115（90.55）	10（50.00）	<0.001
植入时间[d，*M*（范围）]	15（6~31）	12（7~37）	14（12~27）	<0.001
+28 d植入率[%（95% *CI*）]	74.42（73.50~75.34）	88.19（88.02~88.35）	50.00（47.14~52.86）	<0.001
+100 d aGVHD发生率[%（95% *CI*）]				
Ⅰ~Ⅱ度	27.91（26.96~28.85）	24.45（24.15~24.75）	15.00（13.63~16.37）	0.424
Ⅲ~Ⅳ度	7.10（6.78~7.41）	7.26（7.15~7.37）	11.11（9.91~12.31）	0.903
3年cGVHD发生率[%（95% *CI*）]	20.56（19.70~21.42）	31.35（30.86~31.84）	29.63（26.71~32.55）	0.484
3年中重度cGVHD发生率[%（95% *CI*）]	5.04（4.79~5.28）	23.25（22.88~23.61）	12.04（10.59~13.48）	0.024

注 血培阳组：革兰阴性菌血培养阳性；肛拭阳组：肛拭子耐碳青霉烯类革兰阴性菌阳性；双阳组：两项检测结果均阳性；aGVHD：急性移植物抗宿主病；cGVHD：慢性移植物抗宿主病

四、感染/定植的临床特点

63例BSI患者首次感染出现的中位时间为+3 d（−28 d～+11 d），感染最多出现于+1 d。1例（1.59％）患者检测出感染两种菌群。46例（73.02％）患者在粒细胞缺乏（粒缺）期发生BSI。14例（22.22％）患者分离菌株耐药检测为CRE，22例（34.92％）为耐超广谱β内酰胺酶（ESBL）。

147例CRO肛周筛查阳性患者中，24例（16.33％）患者筛查出两种及以上菌群，20例（13.61％）患者同时发生G^−^菌BSI。在此20例患者中，55.00％（11/20）的患者血培养分离菌株耐药检测为CRE，10.00％（2/20）为ESBL，兼有CRE和ESBL 1例（5.00％）。

五、抗感染治疗方案调整

双阳组与血培阳组和肛拭阳组相比，在原有抗感染方案的基础上加用多黏菌素和（或）头孢他啶阿维巴坦（CAZ-AVI）人数最多（*P*<0.001），且加用间隔时间最短（*P*＝0.044）。血培阳组与肛拭阳组和双阳组相比，使用多黏菌素和（或）CAZ-AVI天数最短（*P*＝0.011）。三组患者在感染/定植前是否已使用抗生素进行预防方面差异无统计学意义（*P*＝0.179）（表3）。所有患者均采用标准剂量（多黏菌素：50万IU，每12 h 1次；CAZ-AVI：2.5 g，每8 h 1次）治疗。

**表3:** 造血干细胞移植后微生物检测阳性患者多黏菌素和（或）CAZ-AVI调整比较

临床特征	血培阳组（43例）	肛拭阳组（127例）	双阳组（20例）	*P*值
加用多黏菌素和（或）CAZ-AVI [例（%）]	13（30.23）	47（37.01）	18（90.00）	<0.001
感染/定植后加用间隔时间[d，*M*（范围）]	3（0~49）	10（−12~29）	1（−13~27）	0.044
使用天数[d，*M*（范围）]	4（1~15）	9（1~44）	6（2~23）	0.011
感染/定植前已使用抗生素 [例（%）]	36（83.72）	118（92.91）	18（90.00）	0.179

注 血培阳组：革兰阴性菌血培养阳性；肛拭阳组：肛拭子耐碳青霉烯类革兰阴性菌阳性；双阳组：两项检测结果均阳性；CAZ-AVI：头孢他啶阿维巴坦

六、移植结局

与血培阳组和肛拭阳组相比，双阳组患者的3年DFS和OS率最低（DFS：35.6％对53.7％对68.6％，*P*＝0.001；OS：44.4％对62.4％对76.9％，*P*<0.001），NRM最高（50.0％对34.9％对10.6％，*P*<0.001）（表4）。

**表4:** 造血干细胞移植后微生物检测阳性患者移植结局比较［％（95％*CI*）］

组别	例数	3年				5年			
		CIR	NRM	DFS	OS	CIR	NRM	DFS	OS
血培阳组	43	11.3（10.7~11.9）	34.9（33.7~36.1）	53.7（39.5~72.9）	62.4（48.6~80.0）	17.2（16.0~18.5）	34.9（33.7~36.1）	47.7（32.5~70.0）	62.4（48.6~80.0）
肛拭阳组	127	20.8（20.5~21.1）	10.6（10.5~10.8）	68.6（60.6~77.6）	76.9（69.6~85.0）	20.8（20.5~21.1）	10.6（10.5~10.8）	68.6（60.6~77.6）	76.9（69.6~85.0）
双阳组	20	14.4（12.3~16.6）	50.0（47.4~52.6）	35.6（18.4~68.9）	44.4（27.1~72.9）	14.4（12.3~16.6）	50.0（47.4~52.6）	35.6（18.4~68.9）	44.4（27.1~72.9）
*P*值		0.254	<0.001	0.001	<0.001	0.254	<0.001	0.001	<0.001

注 血培阳组：革兰阴性菌血培养阳性；肛拭阳组：肛拭子耐碳青霉烯类革兰阴性菌阳性；双阳组：两项检测结果均阳性；CIR：累积复发率；NRM：非复发死亡率；DFS：无病生存；OS：总生存

七、影响NRM的因素分析

将性别、年龄、诊断、移植方式、供者类型、CD34^+^细胞输注量、有核细胞输注量、aGVHD和cGVHD的发生情况、造血重建、感染/定植前是否使用抗生素、是否发生BSI、是否粒缺期内发生BSI、是否粒缺期内发生肛周定植纳入单因素分析，结果显示NRM的影响因素为年龄、供者类型、是否发生cGVHD、造血重建、是否发生BSI、是否粒缺期内发生BSI（均*P*<0.05）；多因素分析结果显示，血小板未植入和发生BSI为NRM的独立危险因素（均*P*<0.001）（表5）。

**表5:** 造血干细胞移植后微生物检测阳性患者影响非复发死亡的单因素与多因素分析

临床特征	单因素分析		多因素分析	
	*HR*（95%*CI*）	*P*值	*HR*（95%*CI*）	*P*值
年龄（≥45岁，<45岁）	2.15（1.12~4.11）	0.02	1.43（0.58~3.57）	0.44
移植方式（亲缘半相合，亲缘全相合）	3.67（1.15~11.7）	0.03	3.00（0.64~14.0）	0.16
是否发生慢性移植物抗宿主病	0.27（0.09~0.83）	0.02	0.84（0.23~3.14）	0.80
是否粒系植入	0.03（0.01~0.06）	<0.001	0.28（0.08~1.07）	0.06
是否血小板植入	0.06（0.03~0.12）	<0.001	0.11（0.03~0.36）	<0.001
是否发生血流感染	4.29（2.19~8.39）	<0.001	4.82（1.95~11.9）	<0.001
是否粒细胞缺乏期发生血流感染	2.96（1.57~5.61）	<0.001	0.85（0.36~2.01）	0.71

抗生素调整方面，对63例发生BSI患者行单因素分析，结果显示NRM的影响因素为是否加用多黏菌素和（或）CAZ-AVI及其加用间隔时间与使用天数（均*P*<0.05）；多因素分析结果显示，使用多黏菌素和（或）CAZ-AVI天数>7 d为NRM的独立保护因素（*P*＝0.035）（表6）。

**表6:** 造血干细胞移植后微生物检测阳性中63例血流感染患者影响非复发死亡的单因素与多因素分析

临床特征	单因素分析		多因素分析	
	*HR*（95%*CI*）	*P*值	*HR*（95%*CI*）	*P*值
是否加用多黏菌素和（或）CAZ-AVI	2.76（1.22~6.22）	0.014	0.65（0.16~2.61）	0.540
加用间隔时间（≤7 d，>7 d）	3.21（1.46~7.06）	0.004	2.16（0.66~7.11）	0.200
使用天数（≤7 d，>7 d）	4.25（1.92~9.40）	<0.001	3.73（1.10~12.69）	0.035

注 CAZ-AVI：头孢他啶阿维巴坦

## 讨论

据报道，13％～60％接受HSCT的患者发生BSI，病死率达12％～42％[14]。尤其在粒缺期间，由于免疫能力严重低下，HSCT患者发生感染常较为严重，如不积极预防及治疗，感染相关死亡率很高[15]。因此，加强重点菌群监测、早期识别耐药菌感染、感染后治疗方案的及时调整，对改善血液病患者生存而言至关重要。

我国数据显示，发生BSI的血液病患者分离出的病原菌以大肠埃希菌、肺炎克雷伯菌等G^−^菌为主[16]–[17]。冯丹等[18]发现其所在中心HSCT术后G^−^菌感染中大肠埃希菌占28.2％，肺炎克雷伯菌占12.2％，铜绿假单胞菌占4.5％。在我们的研究中，发生BSI排名前四位的G^−^菌为大肠埃希菌（43.75％），肺炎克雷伯菌（40.63％），铜绿假单胞菌（4.69％）和阴沟肠杆菌（4.69％），这与国内文献报道基本相符。多项研究显示，大肠埃希菌为我国HSCT后BSI最常见菌群，值得重点关注。

在我国，CRE感染中最为常见的菌株之一为肺炎克雷伯菌[19]。我们的研究中，63例BSI患者分离菌株耐药检测显示有14例（22.22％）为CRE，感染菌株最多为耐碳青霉烯肺炎克雷伯菌，检出12株（85.71％）。Zhu等[20]对320例患者进行肛拭子检测，收集到21株CRE菌群，最多为耐碳青霉烯肺炎克雷伯菌（71.43％），其次是耐碳青霉烯大肠杆菌（28.57％）。本研究中，147例CRO肛周定植的患者分离出178株病原菌，耐碳青霉烯肺炎克雷伯菌（32.58％）最多，其次是耐碳青霉烯大肠杆菌（27.53％）。因此，需警惕耐碳青霉烯肺炎克雷伯菌与耐碳青霉烯大肠杆菌感染。已有研究表明，CRE定植是CRE感染的危险因素，且感染菌株多来自患者自身消化道[21]。在本研究中，有20例（13.61％）患者检测出CRO定植且发生G^−^菌BSI。其中70.00％（14/20）患者血培养分离菌株耐药检测为CRE或ESBL。此20例患者的3年DFS和OS率与血培阳组和肛拭阳组比最低，NRM最高。该亚组患者生存差可能与耐药菌BSI发生率高、感染重有关。预后因素分析显示，血小板未植入和发生BSI为影响NRM的独立危险因素（均*P*<0.001）。这提示我们CRE定植并入血引起BSI会严重影响HSCT患者生存，定期持续筛查CRE、采取积极措施预防感染非常必要[7]。

因其高耐药性，CRO感染的临床治疗存在困难。文献显示，CAZ-AVI与多黏菌素类抗菌药可治疗CRO[5]。本研究发现，对于发生BSI的患者而言，使用多黏菌素和（或）CAZ-AVI时间的长短对其NRM的影响有意义，使用天数>7 d为NRM的独立保护因素，这提示我们在临床选择更换抗生素为多黏菌素和（或）CAZ-AVI后，应按一定疗程坚持使用。本研究中，多数患者调整抗生素为多黏菌素和（或）CAZ-AVI时感染已病情危重，因此对于感染发生后何时加用此两种药物能对患者的生存起到改善作用，我们并未得出可靠结果。

本研究的局限性在于这是一项单中心回顾性研究，结果缺乏一定的普遍性，为临床诊疗提供的参考价值有限。

综上所述，对于接受HSCT的患者而言，感染是影响其生存的一大因素。在耐药菌泛滥的如今，早期识别重点菌群在HSCT患者中的定植与感染至关重要。有关CRO感染预警、抗菌药物优化使用方案等，有待更大样本、前瞻性研究验证。
